# Outpatient monthly plasmapheresis with post-PLEX evinacumab in pediatric homozygous familial hypercholesterolemia: a case report on port access and immunoglobulin preservation

**DOI:** 10.3389/fped.2025.1559147

**Published:** 2025-07-18

**Authors:** Guido Filler, Kambiz Norozi

**Affiliations:** ^1^ GF and KN are Professors of Pediatrics and Medicine, Departments of Pediatrics and Medicine, Western University, and Children’s Hospital, London Health Sciences Centre, London, ON, Canada; ^2^ Department of Pediatric Cardiology and Intensive Care Medicine, Hannover Medical School, Hannover, Germany

**Keywords:** homozygous familial hypercholesterolemia, evinacumab, plasmapheresis, pediatric hyperlipidemia, case report

## Abstract

Homozygous familial hypercholesterolemia (HoFH) is a rare genetic disorder characterized by severely elevated low-density lipoprotein (LDL) cholesterol levels, predisposing patients to early cardiovascular disease. While LDL apheresis is the standard extracorporeal therapy, plasmapheresis (PLEX) is often used in younger children due to limitations in vascular access. Evinacumab, an ANGPTL3 inhibitor, has emerged as an effective adjunct for lowering LDL cholesterol. This case, to our knowledge, describes the first pediatric outpatient implementation of monthly plasmapheresis with post-PLEX Evinacumab infusions, enabled by dual-port access and Octaplasma support to maintain immunoglobulin levels. This report highlights procedural innovations that enabled sustained therapy and emphasizes the need for standardized approaches for combining Evinacumab with extracorporeal treatment in pediatric HoFH.

## Introduction

Homozygous familial hypercholesterolemia (HoFH) is caused by biallelic mutations in the LDL receptor (LDLR) gene, resulting in markedly elevated LDL cholesterol and premature atherosclerosis (1). Conventional management includes high-intensity statins, ezetimibe, PCSK9 inhibitors, and extracorporeal LDL cholesterol removal techniques (2). LDL apheresis remains the gold standard, but its implementation is often limited in pediatric populations due to technical and funding challenges, particularly in Canada (3). Plasmapheresis (PLEX) serves as an alternative.

Evinacumab, a fully human monoclonal antibody targeting ANGPTL3, has shown efficacy in significantly reducing LDL cholesterol levels independently of LDL receptor function (4, 5). Investigating the interaction between PLEX and Evinacumab is critical, as PLEX may influence drug clearance by removing circulating monoclonal antibodies, thereby reducing drug bioavailability and potentially diminishing therapeutic efficacy (6). While Evinacumab has been increasingly used in children with HoFH, published data describing its integration with monthly plasmapheresis—particularly in outpatient pediatric settings—are lacking. This case illustrates a practical strategy using dual pediatric ports and Octaplasma supplementation that enabled safe and sustained outpatient therapy.

## Case presentation

• Patient Information: 13-year-old female diagnosed at age 4 with HoFH due to null LDLR mutation (*LDLR* (p.Q349X)).
• Comorbidities: Celiac disease, hypogammaglobulinemia, iron deficiency anemia.
• Family History: Consanguineous parents; sibling deceased due to cardiovascular complications at age 5.
• Medications: Rosuvastatin (20 mg daily), Ezetimibe (10 mg daily), Aspirin (81 mg daily), Vitamin D (1,000 IU daily), Ferrous sulfate (100 mg daily), Evinacumab (650 mg q4w).

## Clinical course

**Figure 1:**
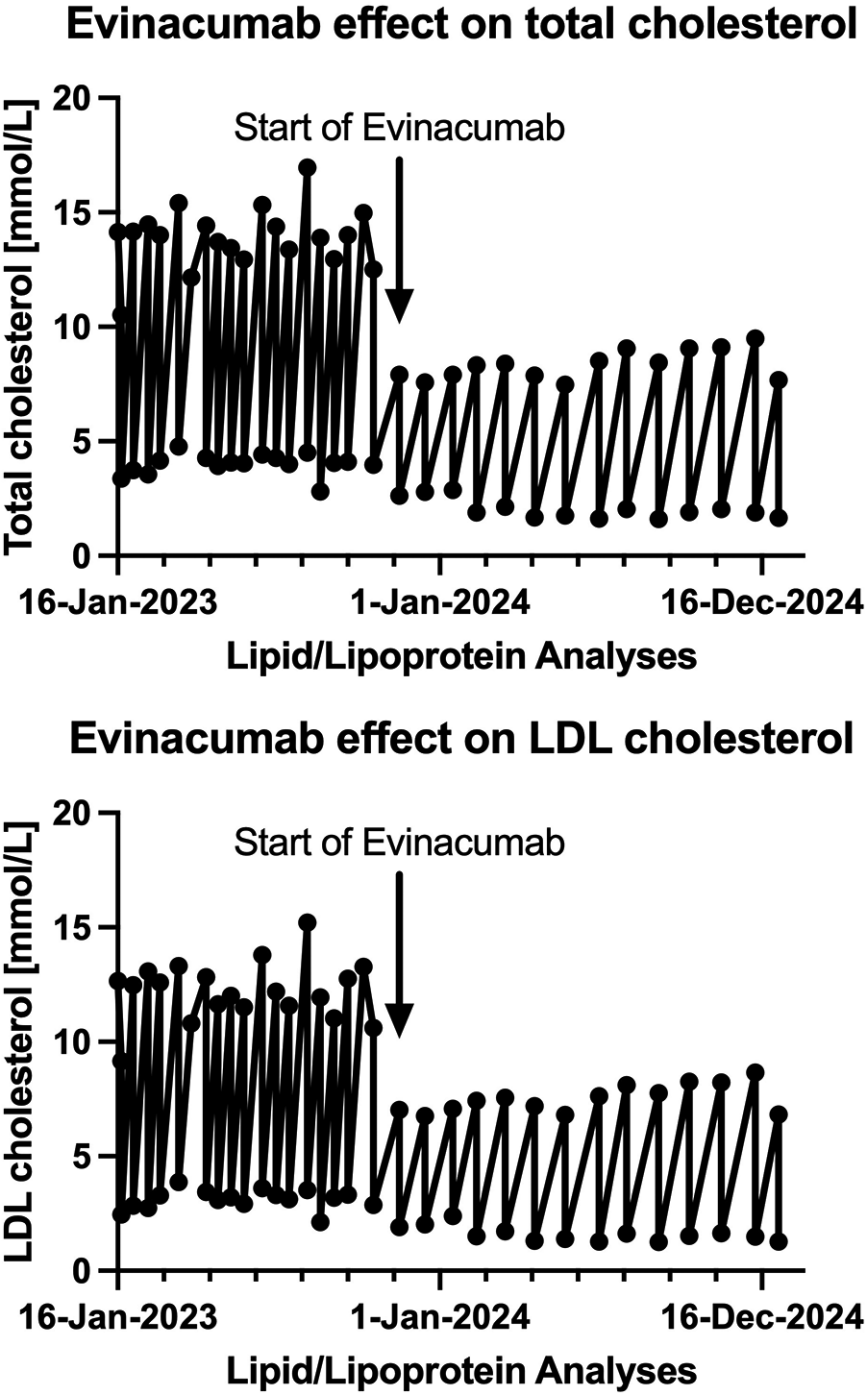
Temporal changes of both total and LDL cholesterol in a now 13-year-old female with hoFH, who was converted from PLEX q 2 weeks to PLEX q 4 weeks and evinacumab infusions after each PLEX treatment.

Initially managed with PLEX every two weeks, she later transitioned to every four weeks with Evinacumab infusions administered post-PLEX. This resulted in improved LDL cholesterol control (1.27 mmol/L, Figure 1). Each PLEX session exchanged approximately 1.0 times the plasma volume over 90 min, using 1,750 ml of 5% albumin followed by 500 ml of Octaplas. Anticoagulation was achieved using Anticoagulant Citrate Dextrose Solution A (ACD-A) at a ratio of 1:12 relative to blood flow. Citrate effects were counteracted with intravenous 10% calcium gluconate, titrated based on symptoms and serum ionized calcium levels.

**Figure 2:**
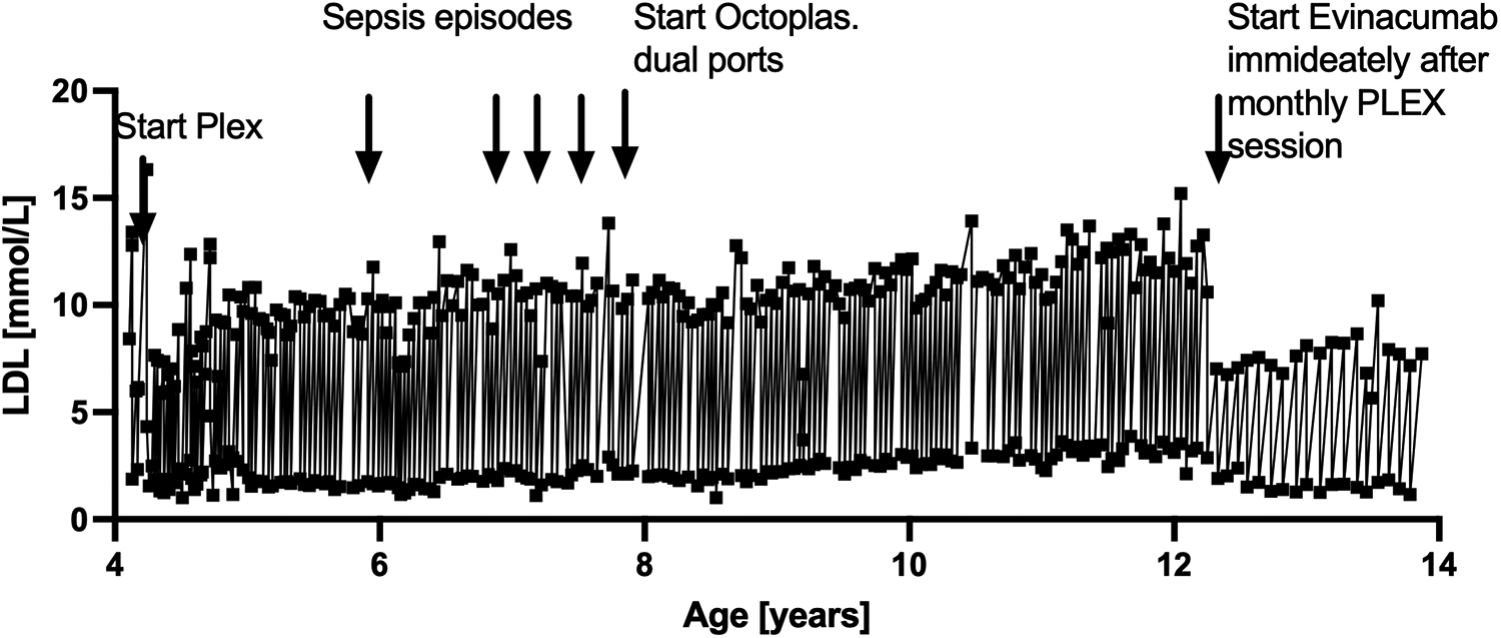
Life-cycle of PLEX treatments now expanded until June 2025 with key events incorporated.

Earlier challenges included recurrent infections due to catheter sepsis and immunoglobulin depletion from PLEX (7). These complications were mitigated by transitioning from percutaneous central venous lines to two single implanted pediatric ports and supplementation with two bags of Octaplasma at the end of each PLEX session. These interventions significantly reduced infection rates and stabilized immunoglobulin levels. The entire lifetime LDL levels are depicted in Figure 2.

## Diagnostics

• LDL cholesterol: 1.27 mmol/L
• Total cholesterol: 1.66 mmol/L
• CBC, renal function: Within normal limits
• Immunoglobulin levels: Maintained >4 g/L using Octaplas during PLEX.

## Therapeutic adjustments

• Optimization of Evinacumab infusion timing post-PLEX
• Pre-medication with H1/H2 blockers for infusion comfort
• Peripheral vein access for enhanced safety
• Continued Octaplasma supplementation to prevent immunoglobulin depletion.

## Outcomes

The patient remained infection-free since the dual pediatric ports and Octaplasma protocol was implemented, and she demonstrated excellent LDL control. Future assessments will evaluate whether PLEX can be phased out in favor of Evinacumab monotherapy. Challenges with transitioning to monotherapy include the unknown durability of LDL cholesterol control without PLEX and the need for long-term data on Evinacumab's efficacy as a standalone therapy.

## Discussion

Evinacumab effectively lowers LDL cholesterol in HoFH patients, irrespective of LDL receptor activity (8). However, PLEX may remove monoclonal antibodies, affecting drug bioavailability and efficacy (9). Administering Evinacumab post-PLEX optimizes exposure but requires careful monitoring. Future pharmacokinetic studies will be essential to determine optimal timing and dosing. In a case report involving a 47-year-old woman with homozygous familial hypercholesterolemia (HoFH), PLEX was used in combination with Evinacumab. However, the report did not provide specific pharmacokinetic data on Evinacumab during TPE (10).

The earlier complications related to vascular access and immunoglobulin depletion, as reported previously (7), highlight the importance of access optimization and plasma supplementation. These findings underscore the need for comprehensive guidelines and further research to optimize therapeutic strategies for HoFH patients. To our knowledge, this is the first pediatric case in which dual implanted ports and Octaplasma supplementation enabled sustained monthly outpatient plasmapheresis combined with post-PLEX Evinacumab therapy. This procedural strategy minimized complications and optimized adherence—an implementation model not previously reported.

Evinacumab represents one of several novel therapies developed for managing homozygous familial hypercholesterolemia (HoFH). Other therapies include the monoclonal antibodies evolocumab and alirocumab, which inhibit proprotein convertase subtilisin/kexin type 9 (PCSK9) to enhance LDL receptor (LDLR) recycling, and inclisiran, a small interfering RNA that reduces PCSK9 synthesis (11). However, these therapies are limited in patients with null LDLR mutations, as they rely entirely on functional LDLRs to lower LDL cholesterol (LDL-C) levels (11). Lomitapide, which inhibits microsomal triglyceride transfer protein to reduce very-low-density lipoprotein (VLDL) assembly and secretion, is effective irrespective of LDLR function but is often limited by significant hepatic and gastrointestinal side effects, restricting its long-term utility (11). Evinacumab's unique LDLR-independent mechanism provides a significant therapeutic advantage for HoFH patients with null mutations. Given the limitations of other monoclonal antibodies dependent on LDLR functionality, Evinacumab should be prioritized in such cases. However, this report's focus on a single patient with limited follow-up highlights the need for further studies to evaluate long-term efficacy, safety, and the feasibility of transitioning to Evinacumab without concomitant PLEX, particularly its impact on growth and development in pediatric populations.

1. Pharmacokinetic studies of Evinacumab with PLEX.
2. Optimal timing and dosing protocols.
3. Long-term outcomes with Evinacumab therapy without concomitant PLEX in pediatric HoFH.

## Patient perspective

The patient's family reported considerable emotional distress during the early stages of treatment, particularly due to frequent hospitalizations for catheter-related infections. The uncertainty around long-term vascular access and repeated intravenous treatments was a constant source of anxiety. They described the transition to implanted dual ports as a major relief—reducing both logistical challenges and the emotional toll of repeated line replacements. The addition of Octaplasma to stabilize immunoglobulin levels further reduced their fear of infections.

Importantly, the family felt actively involved in treatment planning. They expressed appreciation for the transparency regarding the risks and benefits of different therapeutic strategies and the flexibility of outpatient care, which enabled their daughter to attend school more regularly and engage in social activities. The patient herself reported less fatigue and improved confidence due to reduced treatment disruptions and fewer visible medical devices.

## Conclusion

This case demonstrates that monthly plasmapheresis combined with post-PLEX Evinacumab can be feasibly and safely administered in pediatric patients with HoFH using a dual port strategy and Octaplasma-based immunoglobulin preservation. This outpatient approach may offer a sustainable model for long-term care and warrants further study.

### Learning points

1. Evinacumab effectively reduces LDL cholesterol in pediatric HoFH patients undergoing PLEX.
2. Administration timing and infusion protocols are critical for efficacy and safety.
3. Individualized management, including optimized vascular access and immunoglobulin support, is key to minimizing adverse events.

## Data Availability

The original contributions presented in the study are included in the article/Supplementary Material, further inquiries can be directed to the corresponding author.
